# Supplementary material for: Reconfigurable Terahertz Metamaterials Based on the Refractive Index Change of Epitaxial Vanadium Dioxide Films Across the Metal–Insulator Transition
Source: Nanomaterials (Basel). 2025 Mar 13;15(6):439. doi: 10.3390/nano15060439 (PMC11945049; doi:10.3390/nano15060439)
Supplement: Supplementary file 1 [file nanomaterials-15-00439-s001.zip › nanomaterials-3508935-supplementary.pdf]

Supporting Information for

"Reconfigurable Terahertz Metamaterials Based on the Refractive Index Change of Epitaxial Vanadium Dioxide Films Across the Metal-Insulator Transition"

Chang Lu [1], and Weizheng Liang [*2]

1) Department of Electronic Communication and Technology, Shenzhen Institute of Information Technology, Shenzhen 518172, China
2) State Key Laboratory of Featured Metal Materials and Life-cycle Safety for Composite Structures, and School of Resources, Environment and Materials, Guangxi University, Nanning 530004, China

*Correspondence: wzliang@gxu.edu.cn

S1. Film characterization

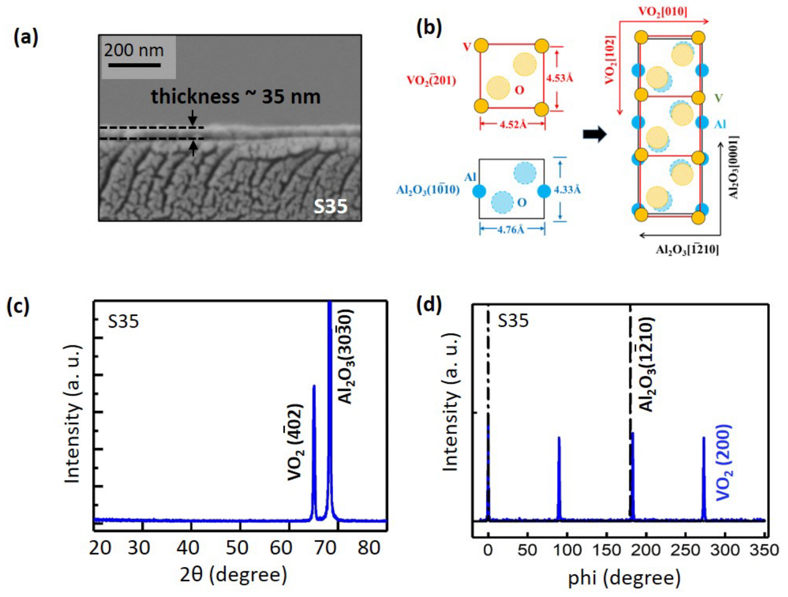

Figure S1. (a) displays scanning electron microscope (SEM) images used to determine the thickness of VO_2 film, which were found to be 35 nm.

Figure S1(b) illustrates the epitaxial relationship between the *m*-sapphire substrate and VO_2 film, which can be summarized as $VO_2(\bar{4}02)$ || $(10\bar{1}0)$ Al_2O_3 in the growth direction and VO_2[102]/[010] || Al_2O_3[0001] in the growth plane. Figure S1(c) shows the room temperature XRD pattern of 35-nm VO_2 film in the $\theta - 2\theta$ scan. The intense $(\bar{4}02)$ reflection at around $2\theta = 65°$ from the $M1$-VO_2 phase confirms the high-quality single-phase VO_2 film. Furthermore, Φ-scan patterns for VO_2(200) diffraction ($2\theta = 37.1^o, \chi = 32.6^o$, blue lines in Figure S1(d), and $(1\bar{2}10)$ Al_2O_3 diffraction ($2\theta = 37.8^o, \chi = 30^o$, black lines) indicate the growth relationship VO_2[102]/[010] || Al_2O_3[0001] in the substrate plane.

Section S2. Lorentz Fitting Examples

The Lorentz fitting process for the simulation results is presented in Figure S2 for two representative cases: (a) n_{THz}= 3, σ_{THz} = 0.1 S/cm and (b) n_{THz} = 30, σ_{THz}= 100 S/cm. Additionally, fitting examples for the experimental results during the heating process is shown in (c) at 54°C and (d) at 60°C. As shown in Figure S2, the Lorentzian peak function accurately fits both the simulated and experimental results, even as the resonances R1 and R2 gradually decay with increased σ_{THz} in (b) and (d). These well-fitting benefits the analysis of resonant properties of ASRR/VO_2 spectra.

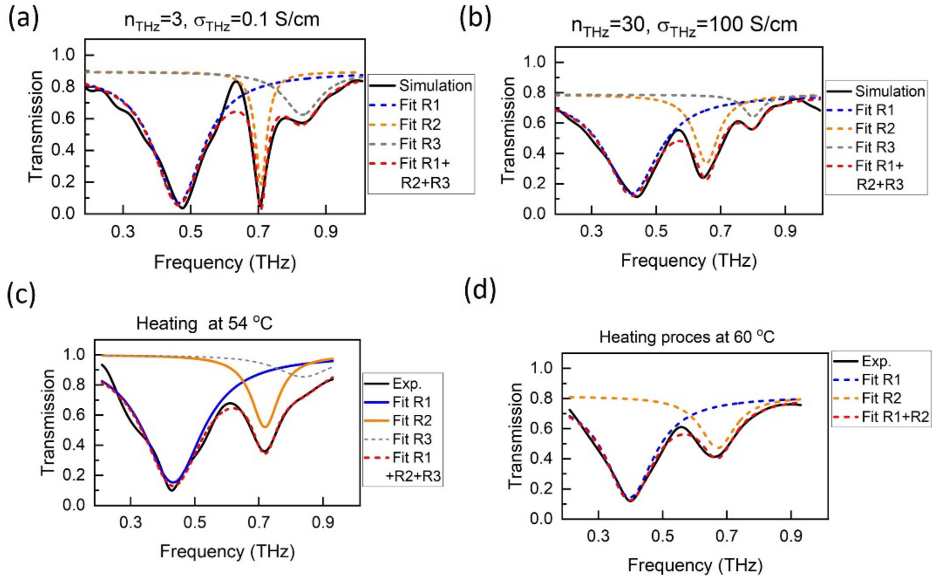

Figure S2. Representative fitting examples of transmittance spectra using Lorentzian resonance functions. (a, b) Fitting results for simulated spectra with *(a)* n_{THz} = 3 and σ_{THz} = 0.1 S/cm, (*b*) n_{THz} = 30 and σ_{THz} = 100 S/cm. (c, d) Fitting results for experimental spectra during the heating process at 54°C (c) and 60°C (d).

Section S3. Simulations for conductivity change in VO_2 film

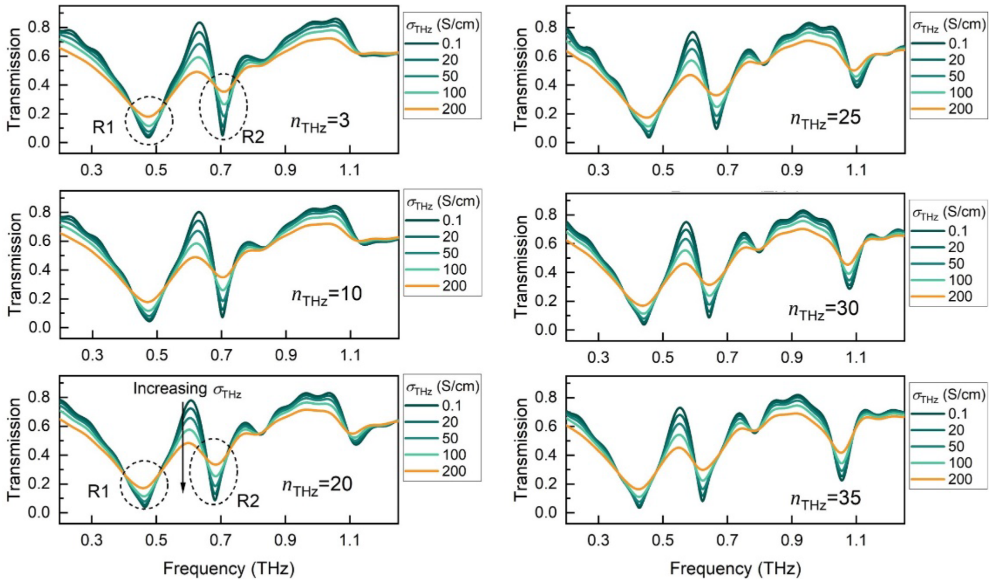

Figure S3. Transmission spectra of ASRR/VO2 at $n_{THz} = 3, 20$, 10, 20, 25, 30, and 35 when σ_{THz} increases from 0.1 to 200 S/cm under TE plane wave (E||x-axis).

Section S4. Time-Domain Signal & data in the cooling process

The time-domain signal shown in Figure S4 (a) presents four representative cases during the heating process, corresponding to the spectra depicted in Figure 4a (main text). As observed, the time-domain signal varies with increasing temperature, which is attributed to the metal-insulator transition (MIT) of the VO_2 film.

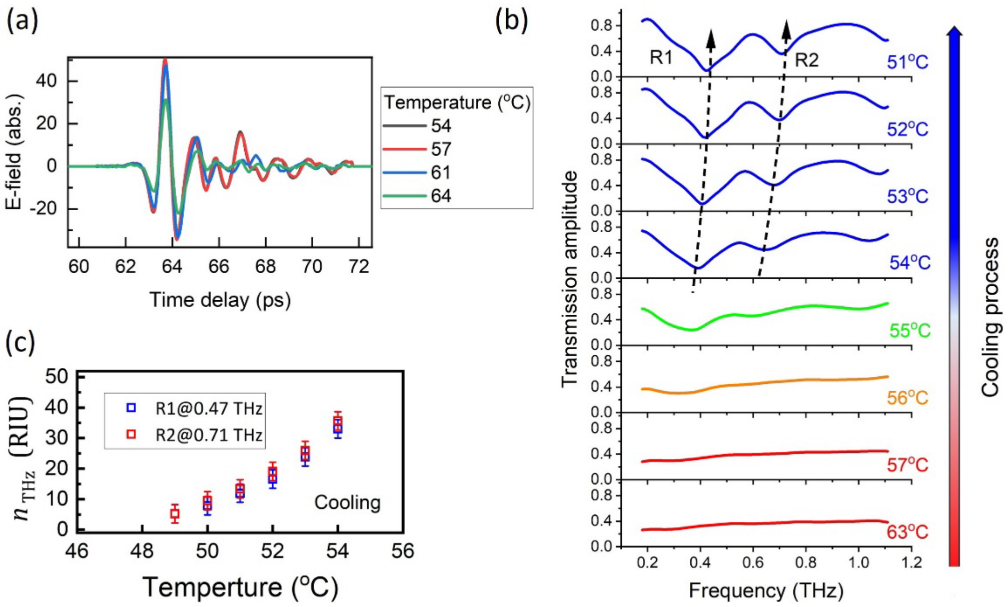

Figure S4. (a) time-domain signals of the ASRR/VO_2 metamaterial during the heating process. (b) Transmission spectra of ASRR/VO_2 as a function of temperature in the cooling process. The n_{THz} extracted from these spectra are shown in (c).

Section S5. THz properties of bare VO_2 film

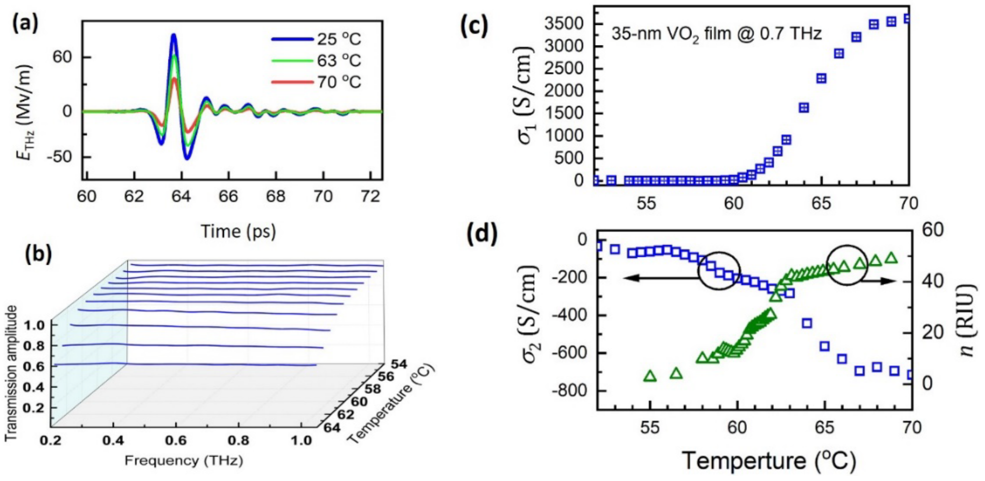

Figure S5. THz properties of VO_2 film measured by THz-TDS. (a) time domain signal; (b) transmission amplitude as a function of temperature; the extracted real-component electrical conductivity σ1 (c), imaginary-component conductivity σ2 (d), and refractive index (d) as a function of temperature.

The time domain signal ($\tilde{t}$) of 35-nm VO_2/ Al_2O_3 measured by THz time domain spectroscopy in transmission setup is shown in Figure S2 (a). Figure S2 (b) gives the transmission spectra of bare VO_2 film, as a result of $|\tilde{t}(\omega)/\tilde{t}_{sub}(\omega)|$, where $\tilde{t}(\omega)$ and $\tilde{t}_{sub}(\omega)$ are the Fourier transformed signal (time domain) of VO_2/ Al_2O_3 and a bare Al_2O_3 substrate, respectively. The complex conductivity ($\tilde{\sigma}(\omega) = \sigma_1 + \mathrm{i}\sigma_2$) of VO_2 film could be extracted from the measured spectra, following the equation:

$$\tilde{t}(\omega)/\tilde{t}_{sub}(\omega)=\frac{1+n_s}{1+n_s+Z_0\tilde{\sigma}(\omega)d}$$

where n_s (set as 3.45) is the refractive index of Al_2O_3 substrate, Z_0 is the free-space impedance, and d (= 35 nm) is the film thickness. The resulted complex conductivity as a function of temperature is shown in Figure S2 (c) in blue squares for the real component, and Figure S2 (d) in blue squares for the imaginary component. The green triangles in Figure S2 (d) represents the refractive index of VO_2 film acquired from imaginary component σ_2, obeying the equation: ${n_{THz}}^2 = \varepsilon_\infty - \sigma_2/\omega\varepsilon_0$.
